# The Burden of Fungal Diseases in Romania

**DOI:** 10.3390/jof4010031

**Published:** 2018-03-01

**Authors:** Mihai Mareș, Valentina Ruxandra Moroti-Constantinescu, David W. Denning

**Affiliations:** 1 Laboratory of Antimicrobial Chemotherapy, Ion Ionescu de la Brad University, 8 Aleea Mihail Sadoveanu, 700489 Iași, Romania; 2 Department of Infectious Diseases, Faculty of Medicine, Carol Davila University of Medicine and Pharmacy, 8 Bulevardul Eroii Sanitari, 050474 Bucharest, Romania; 3 Matei Balș National Institute of Infectious Diseases, 1 Dr. Calistrat Grozovici Street, 021105 Bucharest, Romania; 4 The National Aspergillosis Centre, Wythenshave Hospital, The University of Manchester, Manchester Academic Health Science Centre, Manchester M23 9LT, UK; ddenning@manchester.ac.uk

**Keywords:** mycoses, epidemiology, Romania, candidaemia, aspergillosis, chronic pulmonary conditions

## Abstract

Objective: To estimate for the first time the burden of fungal infections in Romania. Methods: Data derived from the World Health Organization (WHO), National Institute of Statistics, Romanian public health agencies and non-profit health organizations, and published annual reports on local epidemiology were used in the present study. When no data were available, specific at-risk populations were used to calculate frequencies of serious fungal diseases, using previously published epidemiological parameters. All data refer to the year 2016. Results: The estimated number of serious fungal infections in Romanian population was 436,230 in 2016. Recurrent vulvovaginal candidiasis accounted for up to 80% of total cases (more than 350,000 women annually). Concerning HIV-related infections, among 14,349 infected persons, *Pneumocystis* pneumonia occurred in about 10% of late presenters (30 cases in 2016), while cryptococcal meningitis was rarely diagnosed (less than 20 cases). Annually, the total number of oesophageal candidiasis and oral thrush cases in HIV-positive patients may have been as high as 1229 and 3066, respectively. In immunocompromised and cancer patient populations, the annual incidence of candidaemia was 295, and at least 458 invasive aspergillosis cases and 4 mucormycosis cases occurred yearly. With 4966 critical care beds and approximately 200,000 abdominal surgeries performed, the estimated annual incidence of candidaemia and *Candida* peritonitis was 689 and 344, respectively. The annual incidence of pulmonary tuberculosis is still high in Romania (12,747 cases). Thus, the prevalence of post-TB chronic pulmonary aspergillosis is estimated to be 8.98/100,000 (1768 cases). The prevalence of chronic obstructive pulmonary disease (COPD) and asthma in adults is 6% and 6.5%, respectively. Therefore, allergic bronchopulmonary aspergillosis prevalence is estimated at 29,387 and severe asthma with fungal sensitisation at 38,731 cases annually. Conclusions: Not being on the list of reportable diseases, the number of patients presenting with severe mycoses in Romania can only be roughly estimated. Based on local reports and prevalence estimation, we consider that at least 2.23% of Romanians suffer from a serious form of fungal disease.

## 1. Introduction

**Figure 1:**
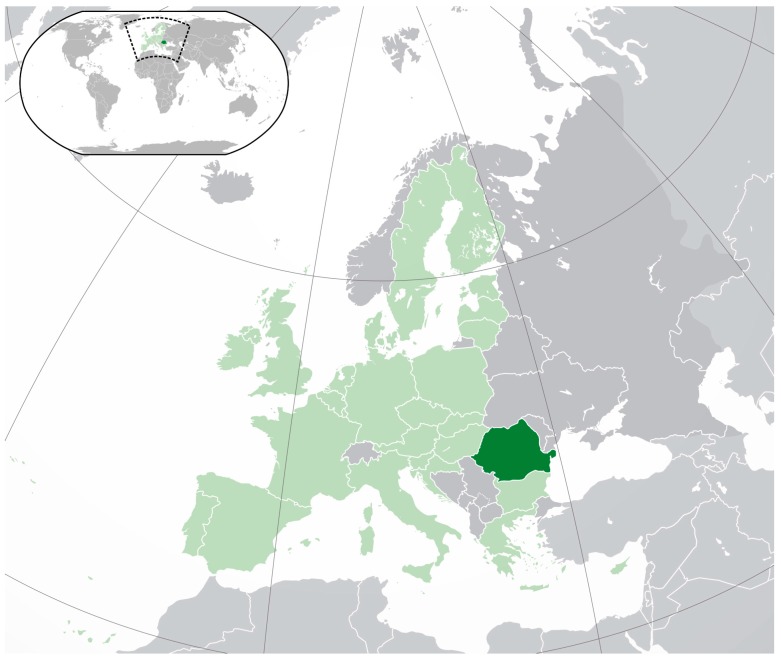
Location of Romania (dark green) in the European Union (green) and Europe (green and dark grey) [1].

Romania is a south-eastern European country, is a member state of the European Union, and shares borders with Hungary to the north-west, Serbia to the south-west, Bulgaria to the south, the Black Sea to the south-east, Ukraine to the east and to the north, and the Republic of Moldova to the east (Figure 1). It covers 238,397 km^2^ (92,046 square miles) and has a population of about 19.6 million inhabitants with a median age of 41.6 years. In Romania, many reports and studies highlight some particular situations that are directly linked with an increased frequency of fungal diseases: high incidence of pulmonary tuberculosis (20% of EU reported cases), more than 1 million cases of chronic obstructive pulmonary disease (COPD), and an important number of HIV-positive patients (about 14,500).

Until now, nationwide data about the incidence and prevalence of serious fungal diseases have never been available in Romania. In light of current efforts being made worldwide to estimate the real importance and implications of fungi in human pathology, this epidemiological study aims to summarize essential information about the incidence and prevalence of serious fungal infections in Romania [2]. It should represent an important starting point, more “pieces of the puzzle” being necessary to better understand the particularities of fungal infections in Romania: species distribution, antifungal susceptibility pattern, emergence of resistance, diagnostic performance in clinical and laboratory settings, potential outbreaks, interventional actions of healthcare authorities, etc. This kind of approach is vital in order to build sustainable solutions to the ongoing problem of invasive and other serious fungal infections alongside numerous chronic conditions which put patients at risk.

## 2. Materials and Methods

Our estimates are based primarily on local data included in national annual reports for 2016. Statistical information concerning the structure of the Romanian population was obtained from the National Institute of Statistics annual reports [3,4].

The total number of adult women possibly diagnosed with recurrent vulvovaginal candidiasis was calculated using the average prevalence of 6%, as previously reported in the literature [5,6].

All data regarding the HIV/AIDS population were derived from the annual report issued by the Compartment for Monitoring and Evaluation of HIV/AIDS Infection in Romania at the *Matei Balș* National Institute of Infectious Diseases [7]. The same report data were considered for the number of *Pneumocystis* pneumonia and cryptococcal meningitis cases that occurred in AIDS [7], whereas the burden of oropharyngeal and oesophageal candidiasis was assessed using incidence data previously published in the literature [8,9,10].

To estimate the number of candidaemia and *Candida* peritonitis cases, the annual incidence of 5/100,000 and 1.5/100,000 respectively were used, in the absence of any national or regional population incidence data [11,12].

Because of the lack of local data, the prevalence of malignant haematological diseases—particularly of acute myeloid leukaemia (AML)—was estimated according to Estey and Dohner [13]. In this group of patients with haematological diseases, the risk of invasive aspergillosis (IA) development was calculated according to Lortholary et al. [14]. The information about all transplantation procedures performed in 2016 was obtained from the National Transplantation Agency [15]. Among patients who underwent haematopoietic stem cell transplantation (HSCT) and solid organ transplantation (SOT), the incidence of invasive aspergillosis and mucormycosis was evaluated according to previous Leading International Fungal Education (LIFE) recommendations [16].

The total number of notified tuberculosis (TB) cases was obtained from the Tuberculosis Surveillance and Monitoring in Europe 2017 report issued by European Centre for Disease Prevention and Control & WHO [17], whereas the estimation of cases of chronic pulmonary aspergillosis (CPA) after TB was done as previously described [18].

The number of patients with COPD and asthma was obtained from the estimative data of the Romanian Society of Pulmonology [19]. The estimated number of patients with allergic bronchopulmonary aspergillosis (ABPA) was calculated as previously described: 2.5% of patients with asthma + 15% of adult patients with cystic fibrosis [20]. The information about cystic fibrosis (CF) prevalence was obtained from the Centre of Monitoring and Treatment of CF at the *Marius Nasta* National Institute of Pneumophthisiology in Bucharest [21].

## 3. Results

In 2016, the population of Romania was 19,679,306 inhabitants, 85.3% of whom were adults and the remainder were children younger than 15 years. Among adults, 8,718,720 were women. The gross domestic product (GDP) per capita was 9520 USD [22]. Table 1 summarizes the burden of different fungal diseases and their incidence or prevalence per 100,000 inhabitants as estimated from our study.

### 3.1. Cryptococcal Meningitis and Pneumocystis Pneumonia

Cryptococcal meningitis and *Pneumocystis* pneumonia (PCP) are among the most severe opportunistic infections occurring in HIV-positive patients and other susceptible groups. The total number of HIV-infected patients was 14,349 (with 654 new cases diagnosed in 2016), 10,942 of whom were receiving antiretroviral therapy (ARV). The crude AIDS-related mortality was 1.30% (189 deaths). The overall incidence of cryptococcal meningitis in Romania was 18 cases per year (0.09/100,000)—12 in HIV-positive patients and 6 in patients presenting other immunosuppresive conditions. The incidence of *Pneumocystis* pneumonia was 0.18/100,000 in 2016, with 30 cases out of 36 diagnosed in HIV patients and the difference occurring in surgery/critical care patients. PCP affects about 10% of late presenters with HIV infection.

### 3.2. Pulmonary Aspergillosis

The number of IA cases was estimated to be as high of 1524 (7.78/100,000). The main underlying conditions in these patients are AML and other malignant haematological diseases, lung cancer, HSCT, and SOT. The prevalence of AML was 3.8/100,000 (748 cases in 2016), whereas the number of HSCT procedures was only 14. Concerning the number of SOT, 295 kidney, 97 liver, and 11 heart procedures were performed in 2016. The prevalence of COPD in adults is about 6% (one million cases), and more than 82,000 patients were hospitalized in 2016. In these COPD patients, an estimated 1066 IA cases occurred (some in intensive care units, ICUs) and 158 in the transplant leukaemia population. The estimated number of IA cases in the lung cancer population was about 300 (2.6%). CPA is diagnosed in patients with chronic pulmonary conditions (i.e., tuberculosis, non-tuberculous mycobacterial infections, COPD). The overall incidence of pulmonary tuberculosis in Romania was 64.77/100,000 (12,747 cases in 2016), 254 of which occurred in HIV-positive patients. TB numbers have been falling in Romania in recent years, but the risk of CPA still remains for some years after pulmonary TB. The annual incidence of new cases of CPA in post TB patients is estimated to be 561 (2.85/100,000). Based on these data, the prevalence of CPA was 1768 after TB and double this overall at 3536 cases (59/100,000).

### 3.3. Mucormycosis

According to our estimates, mucormycosis is a rare fungal infection in Romania, with only seven cases diagnosed in 2016 (0.04/100,000 annual incidence). It occurred in patients with haematological malignancy (four cases) and diabetic patients with ketoacidosis (three cases). Despite its very low incidence, mucormycosis still exhibits a high fatality rate because of late diagnosis and the unavailability of specific therapy (liposomal amphotericin B).

### 3.4. Allergic Bronchopulmonary Diseases

Asthmatic patients may develop ABPA and SAFS. The estimated number of patients with asthma living in Romania is about 1.17 million people. This parameter allowed us to calculate the number of patients with ABPA (29,387) and SAFS (38,731) in 2016. The total number of CF patients was estimated to be as high as 300 in 2016, contributing a maximum of 45 ABPA cases. These asthma-associated diseases are chronic pulmonary conditions, and are among the most difficult respiratory patients to manage optimally. Extra resources are required in order to treat such an important number of patients.

### 3.5. Invasive Candidiasis

In 2016, more than 200,000 abdominal surgical procedures were performed in Romania and many thousands of patients were hospitalized in critical care units across the country, using 4966 beds available in the intensive care units (ICUs). The total number of cases of candidaemia was estimated to 984 (5.00/100,000) in 2016, of which 689 occurred in critical care/surgery settings and 295 in cancer/immunosuppressed patients. This is a conservative population estimate based on other European countries. An important number of ICU patients develop peritoneal candidiasis—344 cases are estimated. Among patients with renal failure undergoing continuous ambulatory peritoneal dialysis (CAPD), there were 50 cases of fungal peritonitis, mainly due to *Candida* species.

### 3.6. Superficial Candidiasis

Among superficial mycoses due to yeasts, recurrent vulvovaginal candidiasis (RVVC) is the most common and problematic illness to be managed in Romania. We estimated that 356,979 women suffer from this disease (3628/100,000 adult females). Oral and/or oesophageal candidiasis are frequent conditions in HIV-positive patients not under ARV. Thus, we assumed that at least 3066 cases of oral candidiasis and 1229 cases of oesophageal candidiasis could be diagnosed annually in these patients. Usually, two or more episodes of oral candidiasis occur each year in these subjects.

## 4. Discussion

The current paper presents for the first time in the literature an epidemiological estimation of fungal diseases in Romania. Although this study represents a rough estimation of the burden of fungal diseases, it can be assumed that at least 3.25 million people (16.5% of the total population) might be affected in Romania—2.23% serious fungal diseases (436,230) plus 14.3% dermatomycoses (2,815,000) [23]. Among these patients, those suffering from difficult-to-treat and potentially life-threatening mycoses represent at least 75,000 each year. The prevalence of superficial fungal infections (dermatomycoses and mucosal infections) could reach 3.17 million cases annually, making mycoses one of the leading causes of morbidity in Romania. Superficial mycoses were actively studied and documented in the literature in the 1960s in Romania, but there has been a 50 year gap in any literature on this topic [24].

There are some particular issues that our study underlines. A precise epidemiological overview in Romania is only possible by putting into practice a prospective multi-annual study. Such a study would also reveal trends, as we suspect that the rate of CPA will be falling slowly as TB cases fall, although the high number of COPD and asthma cases may reverse this (unproven) trend. The annual incidence of opportunistic fungal infections in HIV-positive patients is low, having a similar or even a reduced value compared with other EU countries [25,26]. This is due to the successful implementation of the National HIV/AIDS Programme allowing ARV for the majority of cases. Contrarily, a high incidence of pulmonary tuberculosis occurs in Romania (20% of EU reported cases and an annual rate of more than five times above the EU-28 average). Another important issue is the high prevalence of COPD, with more than 1 million cases in the adult population and more than 80,000 hospitalizations each year. These underlying conditions are risk factors for both invasive and chronic pulmonary fungal diseases, both important causes of increased healthcare expenditures and premature mortality. Likewise, the high rate of lung cancer related to smoking (especially in men) may be contributing to high rates of invasive aspergillosis. The rate of candidaemia was estimated to be 5 cases per 100,000 inhabitants, corresponding to a low average for Europe (3.0–11.4/100,000), higher than Norway and Finland, but significantly lower than Spain, Denmark, and Russia [25,27]. However, this population-based rate needs validation with a registry (as in the UK and Denmark) or a multicentre epidemiological study.

The main drawback of this study is represented by the fact that many of the results are not directly measured prospectively but are products of calculations and formulas. Because these results represent approximations, it is possible that true numbers may differ slightly. However, the estimates are useful as approximations to prioritize both research efforts and healthcare expenditures.

## 5. Conclusions

Our data compilation and analysis have shown that fungal infections are common diseases in Romania and they are rather underestimated because of very few nationwide epidemiological studies. Based on local reports and prevalence estimation, we consider that at least 2.23% of Romanians suffer from a serious form of fungal disease (436,230 cases in 2016). Further epidemiological investigation and surveys are needed in order to obtain realistic data about mycoses-related morbidity and mortality in Romania.

## Figures and Tables

**Table 1:** Serious fungal diseases in Romania.

Fungal Diseases	Rate/100,000	Total Burden
RVVC ^1^	3628	356,979
SAFS ^2^	197	38,731
ABPA ^3^	149	29,387
CPA ^4^	59	3536
Oral candidiasis	16	3066
Invasive aspergillosis	7.7	1524
Oesophageal candidiasis	6.2	1229
Candidaemia	5.00	984
*Candida* peritonitis	1.75	344
Fungal keratitis	1.52	299
Fungal peritonitis in CAPD ^5^	0.25	50
*Tinea capitis*	0.20	40
*Pneumocystis* pneumonia	0.18	36
Cryptococal meningitis	0.09	18
Mucormycosis	0.04	7
Total burden estimated		436,230

^1^ Prevalence of recurrent vulvovaginal candidiasis; ^2^ Prevalence of severe asthma with fungal sensitisation; ^3^ Prevalence of allergic bronchopulmonary aspergillosis; ^4^ Prevalence of chronic pulmonary aspergillosis; ^5^ Annual incidence of fungal peritonitis in continuous ambulatory peritoneal dialysis.
